# Multi-User Identification-Based Eye-Tracking Algorithm Using Position Estimation

**DOI:** 10.3390/s17010041

**Published:** 2016-12-27

**Authors:** Suk-Ju Kang

**Affiliations:** Department of Electronic Engineering, Sogang University, Seoul 04107, Korea; sjkang@sogang.ac.kr; Tel.: +82-2-705-8466

**Keywords:** eye tracking, face detection, multi-user identification

## Abstract

This paper proposes a new multi-user eye-tracking algorithm using position estimation. Conventional eye-tracking algorithms are typically suitable only for a single user, and thereby cannot be used for a multi-user system. Even though they can be used to track the eyes of multiple users, their detection accuracy is low and they cannot identify multiple users individually. The proposed algorithm solves these problems and enhances the detection accuracy. Specifically, the proposed algorithm adopts a classifier to detect faces for the red, green, and blue (RGB) and depth images. Then, it calculates features based on the histogram of the oriented gradient for the detected facial region to identify multiple users, and selects the template that best matches the users from a pre-determined face database. Finally, the proposed algorithm extracts the final eye positions based on anatomical proportions. Simulation results show that the proposed algorithm improved the average F_1_ score by up to 0.490, compared with benchmark algorithms.

## 1. Introduction

**Figure 1:**
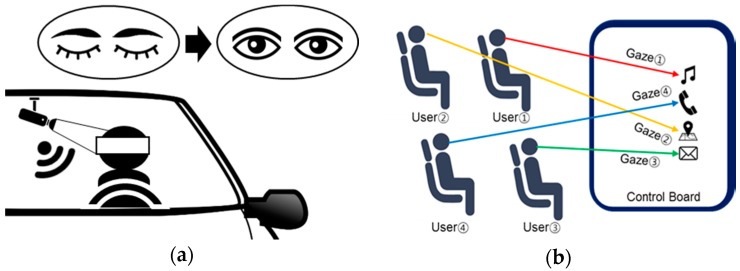
Various examples in a vehicle application: (**a**) a drowsiness warning system; and (**b**) an interface control system using multi-user eye tracking.

Currently, various fields require information about human eye recognition. In particular, the eye recognition is one of the most important features in applications in vehicles because it can estimate human fatigue state, which has a direct impact on the safety of the driver and the passenger. For example, Figure 1a shows a system that checks drowsiness by analyzing the driver’s eyes. In addition, human eyes can be used as an interface to control the operation of the display in the vehicle. Figure 1b shows that the eyes of multiple users control the display of the center console. In these cases, the precise eye positions for multiple users are required. To do so, the eye-tracking algorithm should calculate accurate positional information in the horizontal direction (x), vertical direction (y), and depth direction (z), on the basis of the camera device [1,2].

Various eye-tracking algorithms have been proposed. A video-based eye-tracking algorithm has been proposed [3] to track the eye positions in input frames. This algorithm detects the user’s face using eigenspaces, and estimates motion based on a block-matching algorithm to track the user’s face. However, this algorithm is only suitable for a single user. Another algorithm uses depth and color image sequences for depth-camera–based multi-user eye tracking [4]. This algorithm uses an object-tracking algorithm and eye localization. However, it requires considerable computation time to track multiple users, and it cannot distinguish between them—i.e., it does not associate any particular facial region with a single discrete user.

Generally, eye-tracking algorithms require an accurate face-detection algorithm for high performance. There are two representative face-detection algorithms. A local binary pattern–based algorithm [5,6] uses local image textures in an input image. Hence, it is robust to gray-scale variations, and it is efficient insofar as it uses simple binary patterns. Another approach is a robust real-time face-detection algorithm [7,8]. It uses an integral imaging technique for fast computation. In addition, it uses cascade classifiers based on an adaptive boost-learning algorithm (AdaBoost) to improve the detection accuracy. Eye-tracking algorithms can adopt either of these face-detection algorithms.

In this paper, a new multi-user eye-tracking algorithm is proposed. It is based on a previous study [9], but overall operation blocks are totally changed to enhance performance. The proposed algorithm performs the calibration of red, green, and blue (RGB) and depth images to prevent distortion, and uses the user classification module and several features to enhance the performance. Specifically, it selects the candidate regions (in which faces exist) from an input image. Then, it adopts an AdaBoost-based face-detection algorithm based on [7], and extracts features from the histogram of gradient (HOG) in a facial region. Then, it searches for a template that best matches the input face from a pre-calculated face database. Finally, it estimates and extracts user eye positions based on anatomical proportions.

This paper is organized as follows. Section 2 describes the proposed multi-user eye-tracking algorithm. Section 3 presents performance evaluations comparing the proposal with benchmark algorithms. Section 4 concludes the paper.

## 2. Proposed Algorithm

**Figure 2:**
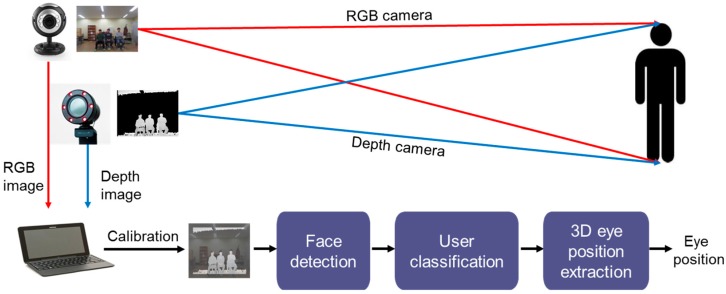
Overall concept for the proposed multi-user eye tracking algorithm.

**Figure 3:**
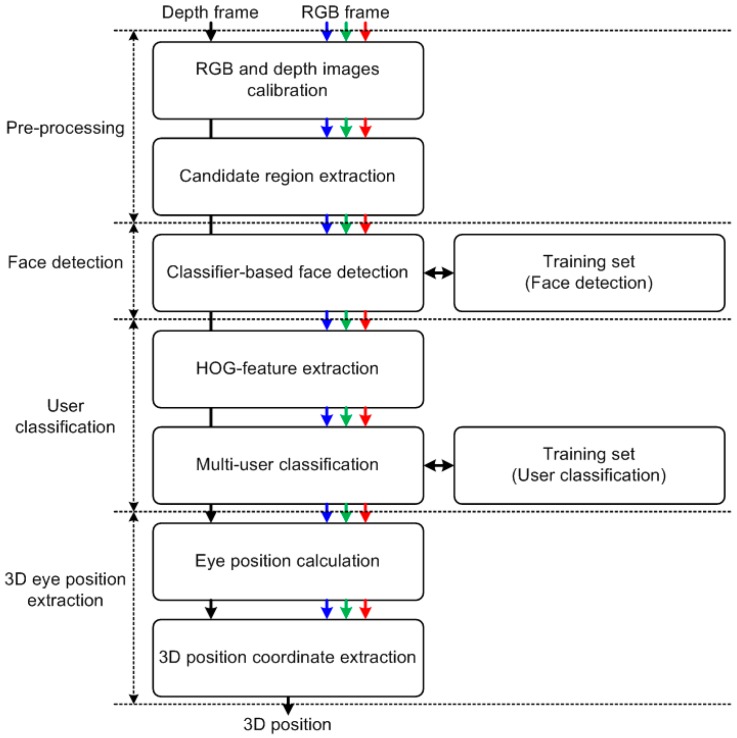
Overall block diagram for the proposed algorithm.

Figure 2 shows a conceptual block diagram for the proposed algorithm. First, in the pre-processing module, the proposed algorithm calibrates the RGB and depth images, which are captured by RGB and depth cameras. Second, the face-detection module performs face extraction from the input images. Third, the user-classification module identifies multiple users. Finally, the 3D eye positions are extracted. Figure 3 shows a detailed block diagram for the proposed algorithm. The specific operations are described in the following sub-sections.

### 2.1. Pre-Processing Module

**Figure 4:**
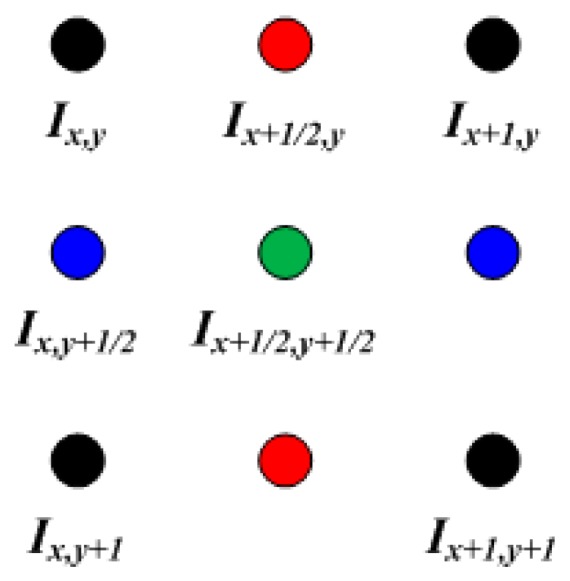
Pixel arrangement in bilinear interpolation algorithm when an input image resolution is doubled.

The proposed algorithm uses RGB and depth cameras. In some cases, the pixel resolution of the RGB and depth images can differ. Hence, the resolutions must be calibrated, and the proposed algorithm increases a low-resolution depth image such that its resolution matches the RGB image. The resolution of depth images is generally lower than that of RGB images. To match the resolution, the proposed algorithm uses a bilinear interpolation algorithm [10], as shown in Figure 4. For example, if the resolution is doubled, it is defined as follows:

(1) Ix+12,y=λ1×{Ix,y+Ix+1,y},Ix,y+12=λ2×{Ix,y+Ix,y+1},Ix+12,y+12=λ3×{Ix,y+Ix+1,y+Ix,y+1+Ix+1,y+1},

where *λ*_1_, *λ*_2_, and *λ*_3_ denote the horizontal, vertical, and diagonal weights, respectively (which are 0.5, 0.5, and 0.25, respectively), and *I_x_*_+1/2,*y*_, *I_x_*_,*y*+1/2_, and *I_x_*_+1/2,*y*+1/2_ denote the horizontal, vertical, and diagonal interpolated pixels, respectively.

Then, the proposed algorithm extracts the candidate search region. In the input image captured by the cameras, the region where users are likely to be when watching a large-sized display such as a television is restricted to a certain area. Therefore, the proposed algorithm uses this region to search for users’ faces, thereby reducing the computation time. The detailed operation for detecting faces is described in the following sub-section.

### 2.2. Face-Detection Module

**Figure 5:**
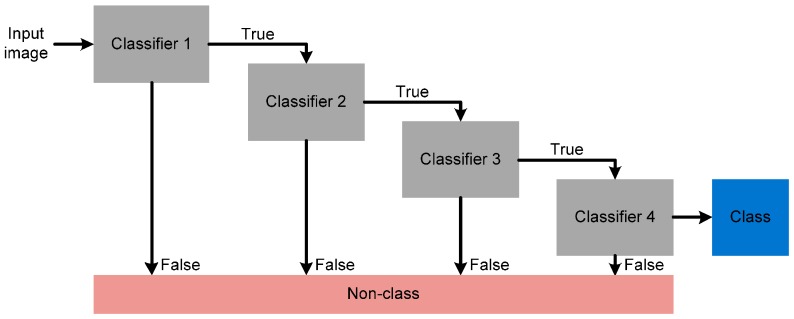
Concept for the cascading structure of the face-detection module in the proposed algorithm.

The proposed algorithm uses the classifier-based face-detection algorithm proposed in [7]. This algorithm offers a high detection rate and it can be operated in real time. In addition, the proposed algorithm analyzes the facial candidate regions selected during pre-processing, thereby enhancing the detection accuracy while reducing the search region. Specifically, the face-detection algorithm uses several rectangular features, and calculates these features based on an integral image [7,11]. This integral image technique generates a summed area table to generate the sum of the pixel values in a rectangular window to enhance the computational efficiency. In addition, it uses simple classifiers generated by the AdaBoost algorithm [7] to select features from the detected face. Finally, the face-detection algorithm uses a cascading structure to generate classifiers which can more accurately detect faces while reducing the operation time. Figure 5 shows the concept for the cascading structure of the face-detection module in the proposed algorithm. The first classifier rejects negative inputs using a few operations. The operations at further stages of the cascade also reject negative inputs, and gradually enhance the accuracy of the detection after multiple stages. Therefore, the proposed algorithm can detect the facial region exactly. 

### 2.3. User-Classification Module

**Figure 6:**
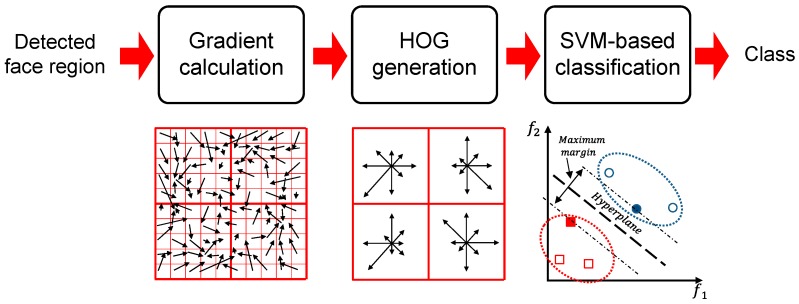
Overall block diagram for the multi-user classification module.

After the faces are detected, they are classified individually based on a pre-calculated database. Figure 6 provides an overall block diagram for this process. The histogram of oriented gradients (HOG) is used as a classification feature because of its robustness in classifying faces [12]. Specifically, the horizontal and vertical gradients for the facial region are calculated as follows:

(2) $$\begin{array}{l} HG=\left[-1\;0\;1\right]*B_{F},\\ VG={\left[-1\;0\;1\right]}^{T}*B_{F}, \end{array}$$

where *HG* and *VG* respectively denote the horizontal and vertical gradients filtered with a 1D-centered discrete derivative mask, and *B_F_* denotes a detected face block. Using the gradients, the HOGs of magnitude and orientation for each pixel are generated as follows:

(3) Mx,y=(HGx,y2+VGx,y2)12,θx,y=tan−1(VGx,yHGx,y)+π2,

where *M_x,y_* and *θ_x,y_* denote the magnitude and orientation of the pixel, respectively. Histograms for the two properties are generated, and histograms for several blocks are combined into one feature vector. Then, the feature vector is classified using a support vector machine (SVM) [13] to partition the classes maximally, thereby generating the exact class for the input face.

### 2.4. Three-Dimensional Eye-Position Extraction Module

**Figure 7:**
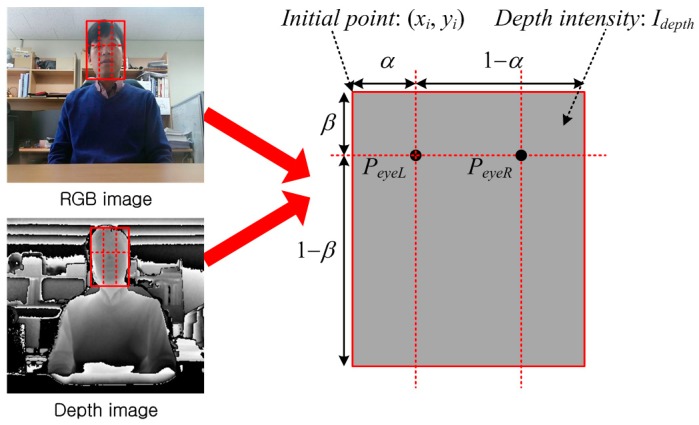
Concept for extracting 3D eye position from the RGB and depth images.

In this module, the proposed algorithm calculates the left and right eye positions. Specifically, it uses the anatomical proportions for the eye position in a human face. Figure 7 shows a conceptual image of this module. First, it computes the horizontal and vertical positions (x and y axes), and then it calculates the depth position (z axis). The image on the left in Figure 7 includes several parameters for calculating the 3D eye position, and these are derived as follows:

(4) $$\begin{array}{l} p_{x1}=x_{i}+\alpha ,\\ p_{x2}=x_{i}+1-\alpha ,\\ p_{y}=y_{i}+\beta ,\\ p_{z}=d_{\max}\times \frac{I_{depth}}{I_{\max}}, \end{array}$$

where *x_i_* and *y_i_* denote an initial pixel point in the detected facial region, *α* and *β* denote the horizontal and vertical offsets, respectively, *I_max_* and *I_depth_* denote the maximum intensity level and the intensity level of the detected face, and *d_max_* denotes the real maximum distance. Using these parameters, the final left and right eye positions are as follows:

(5) $$\begin{array}{l} p_{eyeL}=\left(p_{x1},\;p_{y},\;p_{z}\right),\\ p_{eyeR}=\left(p_{x2},\;p_{y},\;p_{z}\right). \end{array}$$

Using this module, the proposed algorithm can extract the final 3D eye positions.

## 3. Simulation Results

The detection accuracy of the proposed algorithm was evaluated by comparing it with benchmark algorithms. In addition, the identification ratio with multiple users was calculated for the proposed algorithm. The RGB camera had a resolution of 1280 × 960 pixels and the depth camera’s resolution was 640 × 480 pixels. The dataset we used was an image sequence taken with a direct RGB camera and a depth camera in consideration of the distance change. Three benchmark algorithms were used: the classifier-based detection algorithm (Algorithm 1) [7], the improved Haar feature–based detection algorithm (Algorithm 2) [8,9], and the low binary pattern (LBP)-based detection algorithm (Algorithm 3) [6]. For an objective evaluation, the proposed algorithm calculated by precision, recall, and F_1_ scores [14,15], which are derived as follows:

(6) $$\begin{array}{l} \mathrm{Precision}=\frac{TP}{TP+FP},\\ \mathrm{Recall}=\frac{TP}{TP+FN},\\ F_{1}\;\mathrm{Score}\;=2\times \frac{\mathrm{Precision}\times \mathrm{Recall}}{\mathrm{Precision}+\mathrm{Recall}}, \end{array}$$

where *TP*, *FP*, and *FN* denote the number of true positives, false positives, and false negatives that were detected, respectively. Using these values, the F_1_ score was calculated, for which a value of one indicates perfect accuracy. For the test sequences, we used several sequences at different distances (ranging from 1 m to 3.5 m) between the camera and multiple users.

**Figure 8:**
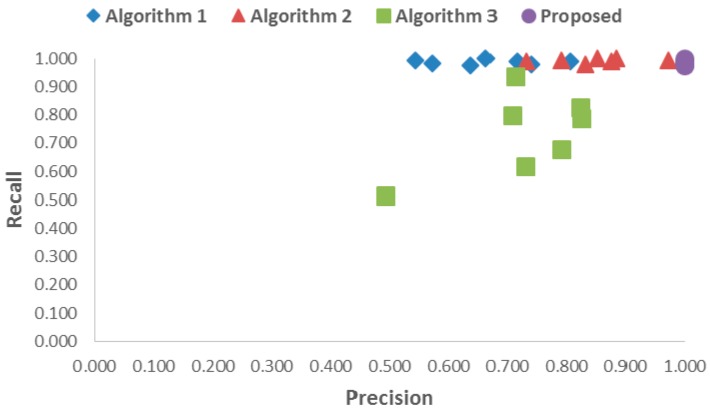
The data distribution of the precision-recall graph for the proposed and benchmark algorithms.

First, the accuracy of detection using the proposed and benchmark algorithms was compared. Table 1 shows the average precision and recall values for the proposed and benchmark algorithms at different distances. Table 2 shows the average F_1_ score, combining precision and recall at different distances. In terms of precision, the total averages of the benchmark Algorithms 1, 2, and 3 were 0.669, 0.849, and 0.726 on average, respectively. In contrast, the proposed algorithm resulted in a perfect score of 1.000. In terms of recall, the total averages of the benchmark Algorithms 1, 2, and 3 were 0.988, 0.993, and 0.738, whereas the proposed algorithm resulted in 0.988. Therefore, the average F_1_ score for the proposed algorithm was up to 0.294, 0.151, and 0.490 higher than those of Algorithms 1, 2, and 3, respectively. This means that the detection accuracy of the proposed algorithm was higher than that of the benchmark algorithms. Figure 8 also shows the same results where the precision and recall values of the proposed algorithm were higher than those of the benchmark algorithms. This was because the proposed algorithm accurately classified foreground and background images by using several cascade classifiers after calibrating RGB and depth images.

**Figure 9:**
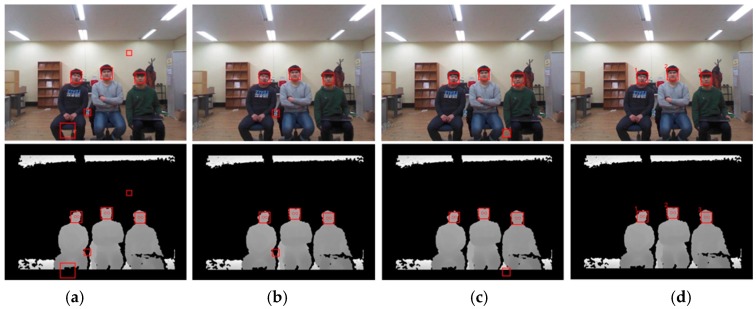
Comparing the detection accuracy of the proposed and benchmark algorithms at a distance of 2.5 m from the RGB and depth cameras (top: RGB image; bottom: depth image): (**a**) Algorithm 1; (**b**) Algorithm 2; (**c**) Algorithm 3; and (**d**) proposed algorithm.

**Figure 10:**
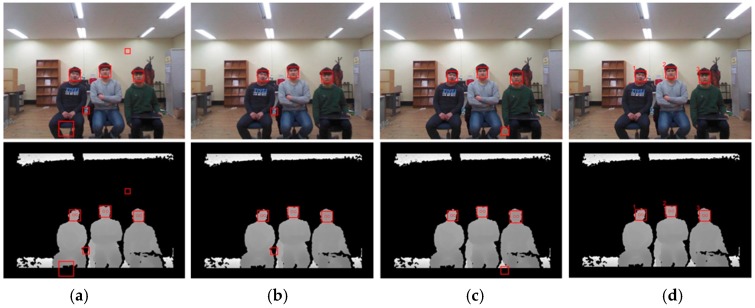
Comparing the detection accuracy of the proposed and benchmark algorithms at a distance of 3.5 m from RGB and depth cameras (top: RGB image; bottom: depth image): (**a**) Algorithm 1; (**b**) Algorithm 2; (**c**) Algorithm 3; and (**d**) proposed algorithm.

Figure 9 and Figure 10 show the resulting RGB and depth images from the proposed and benchmark algorithms at different distances (2.5 m and 3.5 m). The benchmark algorithms detected false regions as faces, and some faces remained undetected. In addition, these algorithms could not associate any particular facial region with a single discrete user. On the other hand, the proposed algorithm accurately detected the faces of multiple users and classified each of them by assigning each face a different number, as shown in Figure 9d and Figure 10d (here, 1, 2, and 3 are the identification numbers for the users).

The identification accuracy of the proposed algorithm for each face from multiple users was also evaluated. Table 3 shows the identification number and ratio for multiple users with the proposed algorithm. The maximum number of users was three. The identification ratios for Faces 1, 2, and 3 were 0.987, 0.985, and 0.997, respectively. In total, the ratio was 0.990 on average, which is highly accurate. This was because the proposed algorithm used the pre-training process for required users, and hence, it had a higher performance than the conventional algorithms.

## 4. Conclusions

This paper presented a robust multi-user eye-tracking algorithm using position estimation. It determines the candidate eye-position regions from input RGB and depth images. Using this region, the proposed algorithm adopts a classifier-based face-detection algorithm, and computes features based on the histogram of oriented gradients for the detected facial region. Then, it selects the template that best matches the input face from a pre-determined database, and extracts the final eye positions based on anatomical proportions. The results of a simulation demonstrated that the proposed algorithm is highly accurate, with an average F_1_ score that was up to 0.490 higher than that of the benchmark algorithms.

## Figures and Tables

**Table 1:** Average precision and recall values for the proposed and benchmark algorithms at different distances.

Distance (m)	Algorithm 1		Algorithm 2		Algorithm 3		Proposed Algorithm	
	Precision	Recall	Precision	Recall	Precision	Recall	Precision	Recall
1.000	0.741	0.981	0.877	0.991	0.730	0.619	1.000	0.981
1.500	0.573	0.985	0.732	0.991	0.493	0.514	1.000	0.985
2.000	0.637	0.975	0.833	0.981	0.825	0.789	1.000	0.975
2.500	0.664	1.000	0.853	1.000	0.713	0.938	1.000	1.000
3.000	0.717	0.991	0.886	1.000	0.824	0.828	1.000	0.991
3.500	0.806	0.991	0.972	0.995	0.708	0.800	1.000	0.991
Random	0.544	0.994	0.792	0.994	0.792	0.677	1.000	0.994

**Table 2:** F_1_ score values for the proposed and benchmark algorithms at different distances.

Distance (m)	Algorithm 1		Algorithm 2		Algorithm 3		Proposed Algorithm
	F_1_ Score	Difference	F_1_ Score	Difference	F_1_ Score	Difference	F_1_ Score
1.000	0.844	−0.147	0.931	−0.060	0.674	−0.320	0.991
1.500	0.725	−0.268	0.842	−0.151	0.503	−0.490	0.993
2.000	0.771	−0.216	0.901	−0.086	0.807	−0.180	0.987
2.500	0.798	−0.202	0.921	−0.079	0.811	−0.189	1.000
3.000	0.832	−0.164	0.939	−0.057	0.826	−0.170	0.996
3.500	0.889	−0.107	0.983	−0.013	0.751	−0.245	0.996
Random	0.703	−0.294	0.882	−0.115	0.731	−0.266	0.997

**Table 3:** Identification number and ratio for multiple users with the proposed algorithm.

Distance (m)	Face 1		Face 2		Face 3	
	Detection Number	Detection Ratio	Detection Number	Detection Ratio	Detection Number	Detection Ratio
1.000	70/70	1.000	68/70	0.970	70/70	1.000
1.500	70/70	1.000	69/70	0.980	69/70	0.980
2.000	64/68	0.940	67/68	0.980	68/68	1.000
2.500	70/70	1.000	70/70	1.000	70/70	1.000
3.000	70/70	1.000	70/70	1.000	70/70	1.000
3.500	69/70	0.980	70/70	1.000	70/70	1.000
Random	89/90	0.990	87/90	0.970	90/90	1.000
